# Supplementary material for: Natural variation reveals that intracellular distribution of ELF3 protein is associated with function in the circadian clock
Source: eLife. 2014 May 27;3:e02206. doi: 10.7554/eLife.02206 (PMC4071560; doi:10.7554/eLife.02206)
Supplement: Supplementary file 1. — RIL periodicity of CCR2::LUC in BxS after photic entrainment. DOI: http://dx.doi.org/10.7554/eLife.02206.021 [file elife02206s001.docx]

**Supplemental Table 1**

**RIL periodicity of *CCR2::LUC* in BxS after photic entrainment**

| **BxS** | **Period LD** | **N** | **SEM** | **# transformants** |  | **BxS** | **Period LD** | **N** | **SEM** | **# transformants** |
| --- | --- | --- | --- | --- | --- | --- | --- | --- | --- | --- |
| 7 | 26.15 | 17 | 0.26 | 2 |  | 146 | 26.00 | 33 | 0.16 | 4 |
| 13 | 26.02 | 23 | 0.15 | 3 |  | 147 | 25.75 | 37 | 0.15 | 3 |
| 15 | 24.11 | 34 | 0.16 | 4 |  | 155 | 26.66 | 21 | 0.27 | 3 |
| 30 | 26.55 | 32 | 0.21 | 3 |  | 157 | 29.58 | 18 | 0.42 | 2 |
| 37 | 26.11 | 12 | 0.30 | 2 |  | 162 | 26.36 | 32 | 0.18 | 4 |
| 45 | 26.69 | 29 | 0.17 | 3 |  | 165 | 26.49 | 34 | 0.15 | 3 |
| 53 | 26.02 | 25 | 0.25 | 4 |  | 173 | 26.28 | 12 | 0.40 | 2 |
| 55 | 26.53 | 17 | 0.49 | 3 |  | 176 | 27.59 | 25 | 0.26 | 3 |
| 59 | 25.16 | 20 | 0.29 | 3 |  | 179 | 27.86 | 33 | 0.15 | 4 |
| 61 | 28.74 | 12 | 0.64 | 2 |  | 183 | 26.79 | 12 | 0.71 | 2 |
| 67 | 27.24 | 26 | 0.46 | 4 |  | 186 | 27.06 | 20 | 0.16 | 4 |
| 70 | 24.87 | 32 | 0.21 | 4 |  | 187 | 26.44 | 20 | 0.20 | 3 |
| 78 | 25.54 | 19 | 0.22 | 3 |  | 190 | 27.45 | 17 | 0.39 | 2 |
| 83 | 25.69 | 20 | 0.34 | 3 |  | 191 | 27.15 | 25 | 0.22 | 4 |
| 92 | 24.55 | 20 | 0.20 | 2 |  | 194 | 25.79 | 16 | 0.25 | 2 |
| 98 | 25.67 | 36 | 0.19 | 4 |  | 195 | 26.49 | 45 | 0.14 | 5 |
| 99 | 26.35 | 17 | 0.48 | 3 |  | 200 | 28.45 | 35 | 0.21 | 4 |
| 102 | 26.62 | 17 | 0.32 | 3 |  | 211 | 27.99 | 25 | 0.33 | 3 |
| 111 | 27.04 | 27 | 0.21 | 4 |  | 214 | 27.91 | 26 | 0.28 | 3 |
| 112 | 25.58 | 20 | 0.33 | 3 |  | 234 | 27.91 | 33 | 0.18 | 4 |
| 114 | 28.22 | 17 | 0.45 | 3 |  | 240 | 28.52 | 26 | 0.30 | 4 |
| 123 | 26.38 | 22 | 0.37 | 3 |  | 252 | 28.25 | 29 | 0.21 | 4 |
| 126 | 25.55 | 12 | 0.41 | 3 |  | 262 | 27.25 | 23 | 0.28 | 3 |
| 127 | 26.71 | 19 | 0.22 | 3 |  | 264 | 27.94 | 17 | 0.33 | 2 |
| 129 | 25.87 | 22 | 0.14 | 3 |  | 298 | 26.12 | 37 | 0.14 | 4 |
| 131 | 25.57 | 18 | 0.27 | 2 |  | 300 | 25.95 | 28 | 0.19 | 3 |
| 133 | 26.26 | 33 | 0.23 | 3 |  | 320 | 27.59 | 20 | 0.22 | 3 |
| 134 | 27.29 | 20 | 0.20 | 3 |  | 325 | 27.07 | 17 | 0.27 | 2 |
| 135 | 26.59 | 43 | 0.14 | 4 |  | 329 | 27.92 | 30 | 0.20 | 5 |
| 136 | 26.58 | 24 | 0.20 | 3 |  | 364 | 26.19 | 37 | 0.39 | 4 |
| 137 | 25.97 | 22 | 0.22 | 3 |  | 368 | 27.31 | 31 | 0.21 | 4 |
| 140 | 27.54 | 38 | 0.14 | 4 |  | 394 | 26.71 | 29 | 0.20 | 3 |
| 143 | 27.83 | 15 | 0.34 | 2 |  | Bay | 27.50 | 19 | 0.45 | 2 |
| Sha | 27.14 | 38 | 0.28 | 3 |  |  |  |  |  |  |

**BxS** denotes RIL number, **Period LD** denotes period in hours after photic entrainment, **N** denotes the individuals assayed per RIL, **SEM** denotes Standard Error of the Mean, **#** denotes number of independent transformants.
