# Supplementary material for: Natural variation reveals that intracellular distribution of ELF3 protein is associated with function in the circadian clock
Source: eLife. 2014 May 27;3:e02206. doi: 10.7554/eLife.02206 (PMC4071560; doi:10.7554/eLife.02206)
Supplement: Supplementary file 2. — RIL periodicity of CCR2::LUC in BxS after thermal entrainment. DOI: http://dx.doi.org/10.7554/eLife.02206.022 [file elife02206s002.docx]

**Supplemental Table 2**

**RIL periodicity of *CCR2::LUC* in BxS after thermal entrainment**

| **BxS** | **Period TMP** | **N** | **SEM** | **# transformants** |  | **BxS** | **Period TMP** | **N** | **SEM** | **# transformants** |
| --- | --- | --- | --- | --- | --- | --- | --- | --- | --- | --- |
| 7 | 25.45 | 21 | 0.27 | 2 |  | 137 | 25.90 | 27 | 0.14 | 3 |
| 13 | 26.17 | 26 | 0.30 | 3 |  | 140 | 27.51 | 42 | 0.12 | 4 |
| 15 | 23.77 | 34 | 0.22 | 4 |  | 143 | 26.65 | 21 | 0.20 | 2 |
| 30 | 26.44 | 38 | 0.17 | 4 |  | 146 | 25.96 | 39 | 0.16 | 4 |
| 37 | 25.48 | 21 | 0.21 | 2 |  | 147 | 25.99 | 40 | 0.18 | 4 |
| 45 | 26.14 | 27 | 0.22 | 3 |  | 155 | 26.56 | 26 | 0.23 | 3 |
| 53 | 26.12 | 32 | 0.17 | 4 |  | 162 | 26.34 | 31 | 0.25 | 4 |
| 55 | 25.27 | 22 | 0.16 | 2 |  | 165 | 26.33 | 26 | 0.23 | 3 |
| 58 | 25.65 | 27 | 0.41 | 3 |  | 173 | 25.65 | 20 | 0.20 | 3 |
| 59 | 24.06 | 25 | 0.27 | 4 |  | 176 | 26.75 | 25 | 0.21 | 3 |
| 61 | 27.08 | 16 | 0.29 | 2 |  | 179 | 26.88 | 33 | 0.16 | 4 |
| 67 | 27.03 | 32 | 0.26 | 4 |  | 186 | 26.20 | 15 | 0.28 | 3 |
| 78 | 25.19 | 33 | 0.18 | 4 |  | 187 | 26.77 | 29 | 0.25 | 4 |
| 81 | 25.94 | 31 | 0.27 | 4 |  | 190 | 25.82 | 19 | 0.24 | 3 |
| 83 | 25.45 | 17 | 0.21 | 3 |  | 191 | 26.20 | 16 | 0.27 | 3 |
| 92 | 24.38 | 22 | 0.20 | 2 |  | 194 | 25.32 | 15 | 0.22 | 2 |
| 98 | 25.54 | 31 | 0.19 | 4 |  | 195 | 26.21 | 38 | 0.17 | 5 |
| 99 | 26.02 | 22 | 0.43 | 3 |  | 198 | 26.79 | 39 | 0.22 | 4 |
| 102 | 27.18 | 30 | 0.34 | 4 |  | 199 | 26.39 | 39 | 0.14 | 4 |
| 106 | 25.94 | 19 | 0.29 | 3 |  | 200 | 27.69 | 34 | 0.21 | 4 |
| 111 | 27.08 | 38 | 0.18 | 4 |  | 211 | 28.11 | 31 | 0.29 | 3 |
| 112 | 25.11 | 24 | 0.24 | 4 |  | 232 | 27.34 | 45 | 0.18 | 4 |
| 114 | 27.62 | 20 | 0.27 | 4 |  | 234 | 26.96 | 37 | 0.18 | 4 |
| 127 | 26.05 | 28 | 0.16 | 3 |  | 240 | 27.00 | 29 | 0.21 | 3 |
| 129 | 25.81 | 27 | 0.15 | 3 |  | 262 | 26.64 | 20 | 0.20 | 3 |
| 131 | 25.15 | 22 | 0.23 | 2 |  | 298 | 25.25 | 38 | 0.12 | 4 |
| 133 | 25.95 | 35 | 0.17 | 3 |  | 300 | 25.17 | 19 | 0.23 | 2 |
| 134 | 27.52 | 20 | 0.26 | 3 |  | 320 | 26.72 | 21 | 0.29 | 3 |
| 135 | 26.19 | 32 | 0.29 | 4 |  | 325 | 26.82 | 12 | 0.21 | 2 |
| 136 | 26.31 | 23 | 0.18 | 3 |  | 329 | 27.09 | 25 | 0.16 | 4 |
| Bay | 25.35 | 30 | 0.31 | 2 |  | Sha | 25.30 | 59 | 0.17 | 4 |

**BxS** denotes RIL number, **Period TMP** denotes period in hours after temperature entrainment, **N** denotes the individuals assayed per RIL, **SEM** denotes Standard Error of the Mean, **#** denotes number of independent transformants.
