# Supplementary material for: Natural variation reveals that intracellular distribution of ELF3 protein is associated with function in the circadian clock
Source: eLife. 2014 May 27;3:e02206. doi: 10.7554/eLife.02206 (PMC4071560; doi:10.7554/eLife.02206)
Supplement: Supplementary file 3. — Detail of primers used. DOI: http://dx.doi.org/10.7554/eLife.02206.023 [file elife02206s003.docx]

**Supplemental Table 3**

**Detail of primers used**

| Name | Type | Sequence Forward | Sequence Reverse | Restriction  Enzyme |
| --- | --- | --- | --- | --- |
| elf3-51 | SSLP | ACTTGATGAAAAACAGATCCAAGAAAAC | AACTTGTCTCAAAATCCTTGAATCTCTC |  |
| elf3-52 | SSLP | AACTTTTCAGTTATGGAAAATTCATTAGA | TTGTGGTTTTCTTGAGTGTGTATGTATG |  |
| elf3-53 | SSLP | AGAGACAGCTCCCTGAAGAGATGAG | TTAAACAAAATTACAAAACTGCCATTTC |  |
| Elf3-54 | SSLP | TTTCAGTTATTGAGATTGACTTAATTTAGTTTT | CGGAACCTAGGTGTTAGCACATTATTTA |  |
| At Elf3 | SSLP | atgatgcccaccataatgaacccaTATTG | aaaggacttgctaccagagattccCTGTG |  |
| elf1001L | CAPS | AAAGACTGACCCAAGAGGATAATG | AGAAGAGTGTGAAGAGAGCCAAAT | *Bam*HI |
| elf1001R | CAPS | TCACGATTTTGACATTTTTAATGG | AAGTGGTGATGGAATTAGATGAGG | *Apo*I |
| U15elf60 | dCAPS | AAGGTTCGGATCCGTATATCT | CTTGAATGGGCCACAAAGAT | *Pst*I |
| U21elf40 | dCAPS | ATGAACCCACAAACTCAGAAGC | CGTCTCTTCCAGATCCATTCTC | *Alu*I |
| U25elf20 | dCAPS | TGGACAACATTCACTGCCTGCA | GCACCCACCACTTGAAAAAT | *Pst*I |
| U35elf60 | dCAPS | GATGGGATCTGGAGGACTCA | AAGTCAATGATGAATTCGTGAGA | *Xmn*I |
| U39elf40 | dCAPS | GTACTCTCTCTGGCTATCATGG | GCACAGTCTCACAAAAGAGCA | *Hae*III |
| U43elf20 | dCAPS | TGCTTCGAAATCTTCTTCTTCTC | GACTCTTTTCCCGACAGCATAC | *Hin*fI |
| U51elf5 | dCAPS | ATCATTCACAAGAGGGTCACTG | TTACTTGGCAATGGCTTTCC | *Pst*I |
| U65elf8 | dCAPS | GTCCAATACGAGTTTGTTTTTA | GGAAGTAAACTTGTAGAGCCATCA | M*se*I |
| Elf3-com |  | gcgcgCCccgggAAaaACccaatAaaaaccacgatcCATTTt | gcgcgccctGcaggTCTTCGTCTCCGCCCTATATTAATCGATGT | *Xma*I/*Sbf*I |
| Elf3-A2V |  | GATTAAGGTTCAAAAACTTATTG**T**TGCATCACCGGATCTCTTGCTCG | CGAGCAAGAGATCCGGTGATGCA**A**CAATAAGTTTTTGAACCTTAATC |  |
| Elf3-V2A |  | GATTAAGGTTCAAAAACTTATTG**C**TGCATCACCGGATCTCTTGCTCG | CGAGCAAGAGATCCGGTGATGCA**G**CAATAAGTTTTTGAACCTTAATC |  |
